# An In Vitro and Ex Vivo Analysis of the Potential of GelMA Hydrogels as a Therapeutic Platform for Preclinical Spinal Cord Injury

**DOI:** 10.1002/adhm.202300951

**Published:** 2023-05-12

**Authors:** Ciara M. Walsh, Jacek K. Wychowaniec, Louise Costello, Dermot F. Brougham, Dearbhaile Dooley

**Affiliations:** ^1^ School of Medicine Health Sciences Centre University College Dublin Belfield Dublin D04 V1W8 Ireland; ^2^ UCD Conway Institute of Biomolecular & Biomedical Research University College Dublin Belfield Dublin D04 V1W8 Ireland; ^3^ School of Chemistry University College Dublin Belfield Dublin D04 V1W8 Ireland; ^4^ AO Research Institute Davos Clavadelerstrasse 8 Davos 7270 Switzerland

**Keywords:** hydrogel, macrophage, microglia, neuroinflammation, organotypic spinal cord slice, spinal cord injury

## Abstract

Spinal cord injury (SCI) is a devastating condition with no curative therapy currently available. Immunomodulation can be applied as a therapeutic strategy to drive alternative immune cell activation and promote a proregenerative injury microenvironment. Locally injected hydrogels carrying immunotherapeutic cargo directly to injured tissue offer an encouraging treatment approach from an immunopharmacological perspective. Gelatin methacrylate (GelMA) hydrogels are promising in this regard, however, detailed analysis on the immunogenicity of GelMA in the specific context of the SCI microenvironment is lacking. Here, the immunogenicity of GelMA hydrogels formulated with a translationally relevant photoinitiator is analyzed in vitro and ex vivo. 3% (*w/v*) GelMA, synthesized from gelatin type‐A, is first identified as the optimal hydrogel formulation based on mechanical properties and cytocompatibility. Additionally, 3% GelMA‐A does not alter the expression profile of key polarization markers in BV2 microglia or RAW264.7 macrophages after 48 h. Finally, it is shown for the first time that 3% GelMA‐A can support the ex vivo culture of primary murine organotypic spinal cord slices for 14 days with no direct effect on glial fibrillary acidic protein (GFAP^+^) astrocyte or ionized calcium‐binding adaptor molecule 1 (Iba‐1^+^) microglia reactivity. This provides evidence that GelMA hydrogels can act as an immunotherapeutic hydrogel‐based platform for preclinical SCI.

## Graphical abstract

Analysis of the immunogenicity of gelatin methacrylate (GelMA) hydrogels in the specific context of the spinal cord injury (SCI) microenvironment is lacking. This article demonstrates the biocompatibility of 3% GelMA‐A in vitro (RAW264.7 macrophages and BV2 microglia) and ex vivo (organotypic spinal cord slices). This paves way for future work exploring GelMA as an immunomodulatory platform for SCI in vivo.

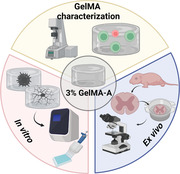

## 1 Introduction

Traumatic SCI is a devastating condition with a rate of 13 to 220 new cases per million people each year, equating to hundreds of thousands of new injuries globally on an annual basis.^[^ ^1^, ^2^, ^3^ ^]^ Despite recent advances in pharmacological and surgical treatment,^[^ ^4^, ^5^, ^6^ ^]^ no curative therapy is currently available. Taken together, this significant unmet clinical need of SCI patients highlights the urgent need for new and effective therapies.

Historically, the SCI preclinical landscape has used in vitro, ex vivo, and in vivo models to unravel the complex multiphasic injury pathophysiology.^[^ ^7^, ^8^, ^9^ ^]^ Lasting for up to 24 h, the “primary injury” phase is initiated by mechanical damage to the spinal cord and results in death of local neural and glial cells.^[^ ^10^, ^11^ ^]^ This triggers the “secondary injury” phase that propagates damage away from the primary injury site and lasts from weeks to months, and as a result is therapeutically targetable. Among the cascade of pathological responses triggered during the secondary injury phase is the disruption of the blood spinal cord barrier (BSCB), and the subsequent invasion and activation of immune cells.^[^ ^9^, ^10^, ^11^ ^]^ The complex inflammatory response is orchestrated by local immune cells, called microglia, and infiltration of circulating neutrophils, macrophages, and T‐ and B‐lymphocytes, to the injury site.^[^ ^12^, ^13^, ^14^ ^]^ Microglia and macrophages at the injury site can have both beneficial and detrimental effects that improve tissue repair or drive further degeneration in a context‐dependent manner that largely depends on their phenotypic state.^[^ ^12^, ^15^, ^16^ ^]^ Traditionally, immune cells were described as existing on a polarization spectrum ranging from a detrimental proinflammatory “M1‐like” state to a neuroprotective anti‐inflammatory “M2‐like” state.^[^ ^12^, ^15^, ^17^, ^18^, ^19^ ^]^ However, this description is overly simplistic, with current focus directed toward establishing a more suitable classification system recognizing the dynamic state of immune cells.^[^ ^20^, ^21^ ^]^ However, traditional classification remains useful for characterizing the complex secondary inflammatory response in vitro, and thus for the purpose of this work we have adopted the terms “M1‐like” and “M2‐like” to simplistically define pro‐ and anti‐inflammatory states, respectively.

The secondary inflammatory response remains an area of therapeutic interest for SCI. Modulation toward an “M2‐like” state by driving alternative activation of immune cells through administration of immunomodulatory molecules and cells can improve functional and histopathological recovery in preclinical studies.^[^ ^17^, ^18^, ^22^, ^23^, ^24^, ^25^ ^]^ However, optimal delivery of therapeutic cells and molecules remains a challenge in SCI, as systemic drug delivery may lead to degradation and low bioavailability at the injury site, while cell grafts often show poor survival upon transplantation into the hostile injury microenvironment.^[^ ^26^, ^27^, ^28^ ^]^ Furthermore, sustained therapeutic delivery is a more appealing approach to target both the onset of inflammation in the acute phase and ongoing inflammation during the sub‐acute and chronic injury phases, but may be difficult to achieve without repeat drug administrations.^[^ ^29^, ^30^ ^]^

Biomaterials are emerging as a promising strategy in terms of therapeutic delivery as they can be introduced directly to the injury site, releasing encapsulated therapeutics gradually through degradation over time, thus acting as a depot for sustained and localized therapeutic release.^[^ ^31^, ^32^, ^33^ ^]^ Additionally, biomaterials can improve cell graft survival and control differentiation.^[^ ^34^, ^35^, ^36^ ^]^ Physical guidance cues can be incorporated to guide axonal regeneration^[^ ^37^, ^38^, ^39^ ^]^ and control immune cell polarization.^[^ ^40^, ^41^, ^42^ ^]^ Hydrogels in particular offer an injectable platform to fill irregularly shaped SCI lesions, with post‐filling in situ polymerization to immobilize or enhance structural fidelity, and tuneable biodegradability for custom therapeutic release profiles.^[^ ^43^, ^44^, ^45^ ^]^ When designing a therapeutic hydrogel, it is important to consider the existence of context‐specific “biocompatible” properties. For example, in SCI applications, hydrogels should; have similar mechanical properties to those of spinal cord tissue with local regions as low as 70–130 Pa;^[^ ^46^, ^47^, ^48^ ^]^ exhibit minimal swelling to avoid increased intraspinal pressure;^[^ ^49^, ^50^ ^]^ and be biodegradable with non‐toxic by‐products.^[^ ^31^, ^32^, ^50^ ^]^ Perhaps most importantly from an immunological perspective, the hydrogel should not exacerbate the existing inflammatory response. Instead, it should be an inert addition to the injury microenvironment, providing structural and trophic support to regenerating tissue through the incorporation of biochemical guidance cues and/or therapeutic cargo.^[^ ^51^, ^52^, ^53^ ^]^

Gelatin is a semi‐synthetic collagen derivative that is widely used as a biomaterial‐based tissue engineering platform. As a hydrolysis product of collagen, one of the major components of the extracellular matrix, gelatin retains cell attachment motifs that support cell growth and survival, lending excellent biocompatibility.^[^ ^54^, ^55^ ^]^ Gelatin can easily be chemically modified to render it photo‐crosslinkable, by reaction of amines on lysine residues with methacrylic anhydride to yield gelatin methacrylate (GelMA).^[^ ^54^, ^56^ ^]^ The methacryloyl groups allow for spatial and temporal control of photopolymerization in the presence of a photoinitiator.^[^ ^56^ ^]^ Furthermore, GelMA yields many advantages over other natural biomaterial counterparts including simple, cost‐efficient, and reproducible production.^[^ ^54^, ^56^ ^]^ This, along with its injectability and biodegradability, has led to GelMA being used for a wide range of biomedical applications including in cell culture,^[^ ^57^, ^58^ ^]^ 3D bioprinting,^[^ ^59^, ^60^ ^]^ tissue engineering,^[^ ^61^, ^62^ ^]^ cell delivery,^[^ ^63^, ^64^ ^]^ and drug delivery.^[^ ^65^, ^66^ ^]^

The biocompatibility, injectability and potential for in situ photopolymerization under mild conditions are particularly appealing for SCI treatment, and as a result, GelMA has been applied in preclinical SCI. These applications mainly relate to cell delivery, e.g., neural stem cells or mesenchymal stem cells,^[^ ^67^, ^68^, ^69^ ^]^ or providing structural support for axonal regeneration.^[^ ^37^, ^70^, ^71^ ^]^ However, these studies focus on functional recovery or axonal regeneration in vivo as their primary outcome and lack a detailed analysis of the direct effect of GelMA on the SCI immune microenvironment. Furthermore, most studies involving GelMA hydrogels in preclinical SCI apply UV radiation‐based photopolymerization, presenting an obvious translational barrier.^[^ ^37^, ^68^, ^69^ ^]^ To act as a successful and translatable immunopharmacological therapy, GelMA hydrogels should not exacerbate tissue damage or amplify the immune response post‐injury, and information relating to the immunological effect of GelMA in the context of SCI is lacking. In this study, we assess the in vitro and ex vivo biocompatibility of GelMA hydrogels formulated with a translationally relevant visible light‐based photoinitiator, with particular focus on the impact of the hydrogel on the SCI/central nervous system (CNS) immune microenvironment to give a better understanding of the potential for GelMA hydrogels as an immunotherapeutic platform for preclinical SCI.

## 2 Experimental Section

### 2.1 GelMA Synthesis

GelMA was prepared as previously described.^[^ ^55^ ^]^ Briefly, 10 g gelatin Type A (G2500, Sigma Aldrich) or Type B (G9382, Sigma Aldrich) was dissolved in 100 mL Dulbecco's phosphate buffered saline (DPBS) at 60 °C. The temperature was dropped to 50 °C and 5 mL of methacrylic anhydride (276685, Sigma Aldrich) was added at a rate of 0.5 mL min^−1^ with gentle mixing. After 1 h, the reaction was quenched by diluting fivefold in 40 °C DPBS. The mixture was then dialyzed against 5 L distilled water using 12–14 kDa dialysis tubing (44146, Serva) for one week at 40 °C under constant stirring, with a full water change once daily. The solution was lyophilized and stored at 4 °C until further use.

### 2.2 ^1^H‐Nuclear Magnetic Resonance (NMR)

The extent of methacrylation was evaluated using ^1^H‐NMR spectroscopy (Varian VnmrS 600 MHz). GelMA and gelatin samples from multiple syntheses were dissolved at 5 mg mL^−1^ in deuterium oxide (Sigma Aldrich) and their spectra were collected at 37 °C using 32 scans with a 30 s relaxation delay. Tetramethylsilane was used as the internal standard for calibrating chemical shift for ^1^H. Phase correction was applied to obtain purely absorptive peaks. Baseline correction was applied before obtaining the areas (integrals) of the peaks of interest. An aromatic resonance from gelatin, at 7.5 ppm, was used as an internal integration standard as it does not take part in the reaction. The degree of methacrylation (DoM) was then determined by comparing the integrated area of the lysine characteristics peak at 2.9 ppm in the GelMA sample with the unreacted gelatin precursor to give a percentage change using the following equation:

(1) $$\mathrm{DoM}\;=\left(1-\frac{\mathrm{Lysine}\;\mathrm{methylene}\;\mathrm{proton}\;\mathrm{of}\;\mathrm{gelMA}}{\mathrm{Lysine}\;\mathrm{methylene}\;\mathrm{proton}\;\mathrm{of}\;\mathrm{gelatin}}\right)\;*100\%$$

DoM is represented as an average ± SD from spectra obtained for three separately synthesized batches. A representative spectrum is provided in Figure S1 (Supporting Information).

### 2.3 Hydrogel Preparation

Hydrogels were prepared by dissolving GelMA powder in 1X PBS at 3, 5, 10, or 15% (*w/v*) at 37 °C with 0.25% (*w/v*) photoinitiator lithium phenyl‐2,4,6‐trimethylbenzoylphosphinate (LAP, 900889, Sigma Aldrich). For sterilization, hydrogel formulations were filtered through a 0.2 µm syringe filter (83.1826.102, Starstedt). Hydrogels were subsequently photopolymerized using a benchtop visible light source for 2 min (power irradiation was determined to be 5.3 W at 1 cm distance from gel). The crosslinking time was selected based on visual inspection of the lowest concentration hydrogel (3% (*w/v*)) at 30 s intervals under the light source until a soft, gel‐like consistency was achieved. This time was then applied for all hydrogel concentrations to maintain the same conditions for each sample. To assess hydrogel injectability, unpolymerized 3% GelMA‐A hydrogel was heated to 37 °C and loaded into a syringe. Once cooled to room temperature, the hydrogel was ejected slowly through a 30 G needle.

### 2.4 Scanning Electron Microscopy (SEM)

GelMA hydrogels were photopolymerized (as described in Section 2.3) in glass scintillation vials and frozen at −80 °C. Hydrogels were then lyophilized and carefully cut into small discs that were coated with a 10 nm layer of gold using Emitech K575X Peltier cooled coater by Quorum Technologies, using a 30 mA sputter current for 15 s. Samples were then imaged using Hitachi Regulus 8230 scanning electron microscope at an acceleration voltage of 3 kV. Pore sizes (diameter) of selected hydrogel samples were manually measured from SEM images using ImageJ (see Figure S2, Supporting Information).

### 2.5 Rheological Measurements

Storage moduli were measured using an Anton Paar MCR301 rheometer fitted with a 25 mm diameter top plate and a 1 mm gap for parallel plate geometry. For sample preparation, 800 µL of hydrogel was photopolymerized in a 12‐well plate. Samples were carefully transferred to the bottom stationary plate of the rheometer using a spatula and the top plate was slowly lowered to minimize sample disruption. The hydrogel was allowed to equilibrate at 37 °C for 3 min prior to each measurement. An amplitude sweep was initially performed for each sample to determine the linear viscoelastic regime (LVR), at 1 Hz and strain increasing from 0.01% to 100% at 37 °C (see Figure S3, Supporting Information). Then, a frequency sweep was measured from 0.1 to 15 Hz at 0.2% strain within the LVR of the hydrogels at 37 °C. The storage modulus values shown represent mean ± SEM from 2–3 independently synthesized GelMA batches, taken from frequency sweeps at *ω* = 6.13 rad s^−1^.

### 2.6 Swelling Assay

Hydrogel samples (100 µL) were photopolymerized in a 1.5 mL tube and 1 mL artificial cerebrospinal fluid (aCSF, 126 mm NaCl, 2.5 mm KCl, 1.25 mm NaH_2_PO_4_, 1.3 mm MgCl_2_.6H_2_O, 26.2 mm NaHCO_3_, 10 mm glucose, 2.5 mm CaCl_2_) was gently added. Samples were incubated at 37 °C. At each time point (0, 1, 3, 7, 14 days), the aCSF was removed and the swollen weight (*W*_wet_) of the hydrogel sample was recorded. Samples were then frozen at −80 °C, lyophilized, and the dry weight (*W*_dry_) was recorded. The swelling ratio was calculated as *W*_wet_/*W*_dry_.

### 2.7 Cell Culture

BV2 microglial cells^[^ ^72^ ^]^ were cultured in Dulbecco's modified Eagle medium (DMEM, LZBE12‐604Q, Lonza) supplemented with 10% fetal bovine serum (FBS, 10270106, Gibco), 1% penicillin and streptomycin (15070‐063, Gibco) and 20 mm HEPES (H0887, Sigma Aldrich). RAW264.7 macrophages^[^ ^73^ ^]^ were cultured in the same medium but without HEPES. Cultures were maintained in a humidified incubator at 37 °C with 5% CO_2_. At 70–90% confluency, cells up to passage 20 were harvested and used for subsequent experiments.

### 2.8 Cytotoxicity Assay

To assess the cytotoxicity of each hydrogel formulation, BV2 cells were collected and resuspended in either complete culture medium or in unpolymerized 3%, 5%, 10%, or 15% GelMA‐A or GelMA‐B solution at a concentration of 75 000 cells mL^−1^. Cell solutions (200 µL) were seeded in a 24‐well glass‐bottomed plate (EP0030741005, Merck) and 500 µL complete cell culture medium was added to each well immediately after photopolymerization as described in Section 2.3. Media was refreshed every 2–3 days. At each timepoint (1, 3, 7 days), 0.2% Triton‐X100 (306324N, BDH Laboratory Supplies Ltd) in DMEM was added to negative control wells and incubated for 5 min at 37 °C. Then, the culture medium from each well was carefully aspirated and replaced with 20 µg mL^−1^ propidium iodide (PI, 2017227, Invitrogen) and 8 µg mL^−1^ fluorescein diacetate (FDA, 343209, Merck) in DMEM to stain dead and live cells, respectively. Plates were returned to the incubator for 15 min, after which cells were washed 3 times with 1X PBS. Plates were immediately imaged using an Olympus FV1000 confocal microscope. The number of live and dead cells were counted using ImageJ, and cell survival is expressed as (#Live cells/#Total cells) × 100. Data are presented as mean ± SEM of 2–3 independent experiments.

### 2.9 Endotoxin Test

Endotoxin levels of gelatin Type A, gelatin Type B, GelMA‐A and GelMA‐B were measured using the Pierce Chromogenic Endotoxin Quant Kit (A39552, Thermo Fisher). Samples were prepared by dissolving each at 3% (*w/v*) in 1X PBS, with 0.25% LAP added for GelMA samples. Sterilization was performed as described in Section 2.3. Under aseptic conditions, 200 µL each sample was added in duplicate to a 24‐well plate. Gelatin was allowed to solidify at room temperature, while GelMA hydrogel samples were polymerized as described in Section 2.3. Then, 1 mL endotoxin‐free water was added to each well and incubated at room temperature for 1 h, after which the sample was harvested and stored at 4 °C overnight. Endotoxin levels were measured according to the manufacturer's instructions, and absorbance values were measured on a SpectraMax Plus 384 spectrophotometer at 405 nm. Final values were calculated using a standard curve of 0.1–1.0 EU mL^−1^ with *R* ^2^ > 0.99.

### 2.10 RNA Isolation and RT‐qPCR

To assess the polarization of macrophages and microglia in 3% GelMA‐A, BV2, or RAW264.7 cells were collected and seeded in a 6‐well plate at a concentration of 2.5 × 10^4^ or 1 × 10^6^ cells per well respectively, in either 3% GelMA‐A or complete culture medium supplemented with 100 ng mL^−1^ lipopolysaccharide (LPS, tlrl‐b5lps, InvivoGen) or 100 ng mL^−1^ interleukin‐13 (IL‐13, 12340135, Immunotools) to stimulate an M1‐like or M2‐like response, respectively. Control cells received no treatment. For RNA isolation, collagenase type I (1 mg mL^−1^, 02195109.3, MP Biomedicals) was added to each well at 24 or 48 h and incubated for 20 min at 37 °C to degrade the hydrogel. RNA was then extracted using the RNeasy Mini Kit according to the manufacturer's instructions (74104, Qiagen) and reverse transcribed with SuperScript II reverse transcriptase (18064014, Thermo Fisher). cDNA was amplified on the Applied Biosystems QuantStudio 7 Flex Real‐Time PCR System using Power SYBR Green technology (4368706, Applied Biosystems) and the primer pairs outlined in **Table** **1**. Quantification was performed using the Δ*Ct* method with PPIA as a housekeeping gene. Gene expression was displayed as fold change relative to control samples, except in the case of Arginase‐1 (Arg‐1) whereby expression was displayed as fold change relative to IL‐13 stimulated samples.

**Table 1:** Primer pairs used for RT‐qPCR

Target	Forward primer (5′–3′)	Reverse primer (5′–3′)
TNF‐*α*	AGCCGATGGGTTGTACCTTG	ATAGCAAATCGGCTGACGGT
iNOS (Nos2)	CAGATCGAGCCCTGGAAGAC	GTGAAGCCATGACCTTTCGC
Arg‐1	GTGAAGAACCCACGGTCTGT	GCCAGAGATGCTTCCAACTG
CD206	GTGGACGCTCTAAGTGCCAT	GAATCTGACACCCAGCGGAA
PPIA	CGTCTCCTTCGAGCTGTTTG	CACCACCCTGGCACATGAAT

### 2.11 Measurement of Cytokine Levels

BV2 and RAW264.7 cells were seeded as described in Section 2.10. Media was collected and replaced at 24 and 48 h to measure cumulative TNF‐*α* and IL‐10 release using the DuoSet ELISA systems according to the manufacturer's instructions (DY410 and DY417, R&D Systems). Absorbance values were measured on a SpectraMax Plus 384 spectrophotometer at 450 nm with a background wavelength of 590 nm.

### 2.12 Preparation of Hydrogels for Ex Vivo Culture

500 µL pre‐warmed sterile hydrogel was added to 0.4 µm polyester (PET) cell culture inserts (83.3930.300, Starstedt) and photopolymerized as described in Section 2.3. Hydrogels were washed and left overnight at 4 °C in sterile 1X PBS to allow for swelling. Hydrogels were then incubated overnight at 4 °C in slice culture media (50% minimum essential media [M4655, Sigma Aldrich], 25% HBSS [H8264, Sigma Aldrich], 25% horse serum [H1270, Merck], 20 mm HEPES [H0887, Sigma Aldrich], 6 mg mL^−1^ glucose [25‐037‐CI, Corning], 1% penicillin/streptomycin [15070‐063, Gibco]). This was removed on isolation day and PET inserts both with and without hydrogels were pre equilibrated in fresh culture medium at 37 °C/5% CO_2_ for at least 30 min before slices were added.

### 2.13 Organotypic Spinal Cord Slice Isolation and Culture

All housing and surgical procedures in this study were approved by the Animal Research Ethics Committee (AREC) at University College Dublin and the Health Products Regulatory Authority of Ireland in accordance with the European Union Directive 2010/63/EU and S.I. No. 543 of 2012. Postnatal day (P) 5–6 C57BL/6 mouse pups (*n*  =  6) were humanely killed by decapitation and the spinal cord was immediately isolated. Briefly, skin and visible muscle was removed from the dorsum and the entire posterior column was removed and transferred to a fresh petri dish. The dorsal portion of the spinal column was removed under a stereomicroscope using spring scissors to expose the spinal cord, which was then gently removed using a fine spatula and transferred to a fresh petri dish with ice‐cold HBSS supplemented with 6 mg mL^−1^ glucose. The meninges were gently dissected away using fine forceps and 350 µm transverse slices were cut using a McIlwain tissue chopper. Remaining steps were carried out in a biosafety cabinet under aseptic conditions. Whole, intact thoracic slices were transferred to prepared 0.4 µm PET inserts, removing any residual liquid from around the slice using a Pasteur pipette. Inserts were placed in a 6‐well plate with 1 mL culture media and cultured at 37 °C/5% CO_2_ using the air–liquid interface culture method. A full media change was performed after 24 h and every 2–3 days thereafter.

### 2.14 Immunohistochemical Staining and Analysis

At 7 and 14 days in culture, slices were fixed with 4% paraformaldehyde (PFA) for 1 h. Immunofluorescent staining was performed to visualize astrocytes (GFAP^+^, G3893, Sigma Aldrich, 1:500) and microglia (Iba‐1^+^, 019‐19741, Wako, 1:250). PET membranes were cut from the plastic insert using a scalpel, and adherent slices were transferred to a 24‐well plate for staining. Slices were blocked and permeabilized for 1 h in 1X PBS with 5% protein block (ab64226, Abcam) and 0.1% Triton‐X100. Slices were incubated in primary antibodies overnight at 4 °C, washed 3 times in 1X PBS and incubated in secondary antibodies for 2 h at room temperature. Secondary antibodies were goat anti‐mouse Alexa Fluor 488 (A32723, Invitrogen, 1:250) and goat anti‐rabbit Alexa Fluor 568 (A11004, Invitrogen, 1:250). Following secondary antibody incubation, slices were washed 3 times and counterstained with 300 nm Hoescht 33342 for 30 min at room temperature, followed by 3 washes with PBS and once with distilled water. Slices were inverted and mounted onto 35 mm glass bottomed dishes (P35G‐1.5‐20‐C, MatTek) with Fluoromount mounting medium (F4680, Sigma Aldrich).

For intensity and area analysis, one Z‐stack was taken per spinal cord slice with a 5 µm step size from the top to the bottom of the slice to capture all emitted fluorescence using an Olympus FV3000 confocal microscope equipped with 405, 488, and 561 nm lasers and a 10× objective lens. For intensity analysis, integrated fluorescent density of a sum projection within the slice ROI was quantified using ImageJ and non‐specific fluorescence was subtracted based on negative controls. For microglial morphological analysis, 2 non‐overlapping Z‐stacks were taken per spinal cord slice with a 2 µm step size and a 40× lens. ImageJ was used to measure cell perimeter, Feret's maximum diameter and Transformation Index. Feret's maximum diameter was defined as the maximum distance between two points of a cell, while transformation index was defined as *Perimeter* ^2^/4*πarea*.^[^ ^74^ ^]^ To exclude noise and aggregated/clumped cells, only cells with an area of 20–400 µm^2^ and a perimeter of 37–300 µm were analyzed.

### 2.15 Statistical Analysis

All statistical analyses were performed using GraphPad Prism 8.0 software. Data were tested for normality using the Shapiro‐Wilk test. BV2 survival data and immunofluorescence data were analyzed by two‐way ANOVA with Tukey's correction for multiple comparison. qPCR and ELISA data were analyzed by one‐way ANOVA with Dunn's correction for multiple comparisons. Differences were considered statistically significant when *p* < 0.05. Data are presented as mean ± SEM.

## 3 Results

### 3.1 GelMA Hydrogel Characterization

**Figure 1:**
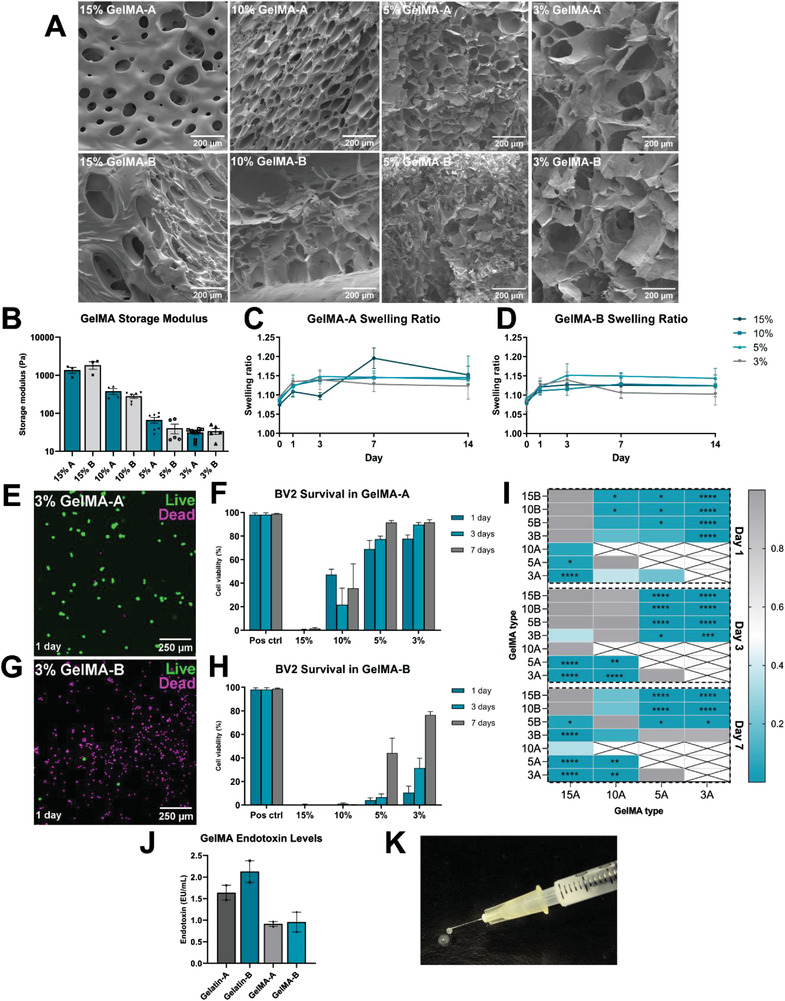
Hydrogel characterization. A) SEM images of 3–15% GelMA‐A and GelMA‐B hydrogels. B) Storage modulus of 3–15% GelMA‐A and GelMA‐B hydrogels. Swelling ratios of 3–15% C) GelMA‐A and D) GelMA‐B. Data presented as mean ± SEM of 2–3 independently synthesized GelMA batches. Immunofluorescent images of live (green, fluorescein diacetate) and dead (magenta, propidium iodide) BV2 cells in 3% E) GelMA‐A and G) GelMA‐B after 1 day in culture, F,H) with quantification of cell survival at each timepoint over 7 days. I) Results of 2‐way ANOVA with Tukey's multiple comparison test on BV2 survival data, comparing BV2 survival in each gel type at each timepoint. Data presented as mean ± SEM of 2–3 independent experiments. J) Endotoxin levels in gelatin Type A and Type B, and the corresponding 3% GelMA hydrogels. Data presented as mean ± SEM of duplicate samples from a single gelatin or GelMA batch. K) Injectability of unpolymerized 3% GelMA‐A hydrogel through a 30 G needle at room temperature. *p < 0.05, **p < 0.01, ***p < 0.001, ****p < 0.0001.

^1^H‐NMR showed an average DoM for the synthesized GelMA of 67.3 ± 1.9% (Figure S1, Supporting Information), which was reproducible across multiple synthesized batches for both gelatin type‐A and type‐B. SEM imaging of the GelMA hydrogels revealed a porous hydrogel microstructure, and pore size analysis showed an expected trend toward increasing pore size with decreasing GelMA concentration (**Figure** **1**A and Figure S2, Supporting Information). Storage moduli of 3–15% (*w/v*) GelMA were measured to assess hydrogel stiffness (Figure 1B). As expected, stiffness decreased with decreasing GelMA concentration, and there was no significant difference between GelMA‐A and GelMA‐B hydrogels of matching concentrations. To assess the extent of swelling of GelMA hydrogels over 14 days, the ratio of the swollen wet weight of each hydrogel to the dried weight was measured at 1, 3, 7, and 14 days (Figure 1C,D). Hydrogels tended to reach a maximum swelling capacity by day 3, after which point no further swelling occurred.

Viability of BV2 microglia cultured within each hydrogel was assessed using FDA and PI to stain live and dead cells, respectively (Figure 1E–H). BV2 cell survival was virtually null in 15% GelMA‐A, 15% GelMA‐B, and 10% GelMA‐B, while survival remained below 50% in 10% GelMA‐A over 7 days (Figure 1F,H). In 5% and 3% GelMA‐B hydrogels, BV2 viability was low on day 1 (4.1 ± 2.7% and 10.6 ± 8.2%, respectively) and improved to 46.3 ± 15.0% and 76.5 ± 2.5% by day 7 (Figure 1H). In contrast, cell viability in 5% and 3% GelMA‐A on day 1 was 46.0 ± 24.0% and 77.8 ± 4.1%, respectively. Viability further improved to 91.5 ± 1.9% and 91.7 ± 3.0% after 7 days (Figure 1F). Upon analysis, cell survival in 3% GelMA‐A was significantly higher than cell survival in each GelMA‐B hydrogel at every timepoint, except at day 7 which showed no significant difference to 3% GelMA‐B (Figure 1I). Taken together, these data suggest that softer (5% and 3%) GelMA‐A hydrogels are less cytotoxic than GelMA‐B hydrogels, with 3% GelMA‐A offering a softer hydrogel platform than 5% GelMA‐A (31.4 ± 4.6 Pa vs 62.0 ± 16.6 Pa, Figure 1B).

Endotoxins, e.g., LPS, are found in the outer membrane of gram‐negative bacteria and can be toxic to neural cells.^[^ ^75^, ^76^ ^]^ The Food and Drug Administration (FDA) dictates that the upper endotoxin limit for clinical medical devices is 0.5 EU mL^−1^.^[^ ^77^ ^]^ To assess if endotoxin content was a contributing factor to the differential survival of microglia in GelMA‐A versus GelMA‐B, we compared the endotoxin content of 3% GelMA‐A, 3% GelMA‐B, and the original batches of unreacted gelatin A and B (Figure 1J). There was no difference between the endotoxin content of GelMA‐A and GelMA‐B (0.92 ± 0.06 and 0.96 ± 0.23 EU mL^−1^, respectively), suggesting that the endotoxin content of each hydrogel was not a contributing factor to the differential microglial survival rate. Furthermore, the synthesized GelMA samples showed lower endotoxin levels than the corresponding gelatin batch, indicating additional endotoxins are not introduced during synthesis. However, we acknowledge that the endotoxin results presented here exceed the current FDA limit and may impact subsequent results describing the immunogenic properties of GelMA.

As a final characterization step, we assessed the injectability of 3% GelMA‐A at room temperature through a 30 G needle, as hydrogel injectability is a key factor for in vivo applications. As seen in Figure 1K, well‐defined droplets extruded well without any needle blockages, indicating the translational potential. Due to good injectability, high cytocompatibility, low stiffness and limited hydrogel swelling, we selected 3% GelMA‐A as the optimal hydrogel formulation for subsequent work.

### 3.2 3% GelMA‐A Does Not Induce an Inflammatory Response in BV2 Microglia at 24 or 48 h in Culture

**Figure 2:**
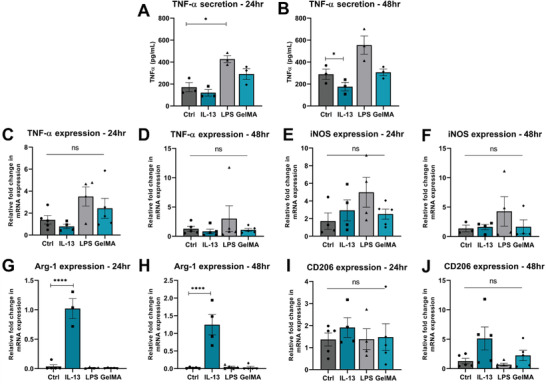
3% GelMA‐A does not significantly affect the polarization of BV2 microglia over 48 h. A,B) TNF‐*α* secretion from BV2 microglia stimulated with 100 ng mL^−1^ IL‐13 or LPS, or grown in 3% GelMA‐A were measured by ELISA at 24 and 48 h. C,D) TNF‐*α*, E,F) iNOS, G,H) Arg‐1, and I,J) CD206 gene expression was measured by qPCR. Gene expression is represented as fold change compared to control using PPIA as a housekeeping gene, with the exception of Arg‐1 expression whereby data are shown as fold change compared to IL‐13 stimulated cells. Data represent mean ± SEM of 3–5 independent experiments. Analysis by one‐way ANOVA with Dunnett's multiple comparisons test, *p < 0.05, ****p < 0.0001, ns *p* > 0.05 compared to control.

Next, we aimed to determine the immunogenicity of 3% GelMA‐A using BV2 microglia, an immortalized cell line offering an in vitro representation of the resident CNS immune cells. BV2 cells stimulated with 100 ng mL^−1^ LPS or 100 ng mL^−1^ IL‐13 acted as an indicator of cell polarization toward “M1‐like” or “M2‐like” polarization states, respectively. The secretion of TNF‐*α* and IL‐10, and the mRNA expression of TNF‐*α*, iNOS, CD206, and Arg‐1 were measured to determine microglial polarization. These markers were selected as basic proxies of cell polarization due to their widespread use in the literature.^[^ ^15^, ^22^, ^78^, ^79^ ^]^ As expected, TNF‐*α* secretion was significantly increased in LPS‐stimulated cells at 24 h (*p* < 0.05, **Figure** **2**A), and significantly decreased at 48 h in IL‐13‐stimulated cells (*p* < 0.05, Figure 2B). There was no detectable IL‐10 secretion from BV2 microglia in any group (data not shown) indicating IL‐10 secretion was below the limit of detection of 31.2 pg mL^−1^. Arg‐1 expression was significantly increased in IL‐13‐stimulated cells, as Arg‐1 is a typical M2‐like marker (*p* < 0.0001, Figure 2G,H). Importantly, at 24 or 48 h in culture, there was no significant difference in the secretion or expression of selected polarization markers between control cells and cells grown in 3% GelMA‐A (Figure 2). Given these results, the measured endotoxin levels of 3% GelMA‐A (Figure 1J) do not appear to negatively contribute to the polarization state of BV2 microglia.

### 3.3 3% GelMA‐A Does Not Induce an Inflammatory Response in RAW264.7 Macrophages at 48 h in Culture

**Figure 3:**
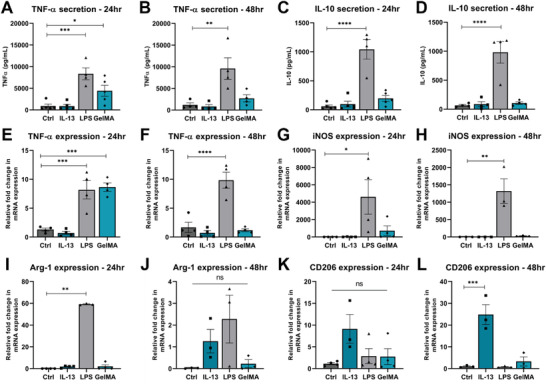
3% GelMA‐A does not significantly affect the polarization of RAW264.7 macrophages at 48 h. A,B) TNF‐*α* and C,D) IL‐10 secretion from RAW264.7 macrophages stimulated with 100 ng mL^−1^ IL‐13 or LPS, or grown in 3% GelMA‐A were measured by ELISA at 24 and 48 h. E,F) TNF‐*α*, G,H) iNOS, I,J) CD206, and K,L) Arg‐1 gene expression was measured by qPCR. Gene expression is represented as fold change compared to control using PPIA as a housekeeping gene, with the exception of Arg‐1 expression whereby data are shown as fold change compared to IL‐13 stimulated cells. Data presented as mean ± SEM of 3–5 independent experiments. Analysis by one‐way ANOVA with Dunnett's multiple comparisons test, *p < 0.05, **p < 0.01, ***p < 0.001, ****p < 0.0001, ns *p* > 0.05 compared to control.

Similarly, we determined the immunogenicity of 3% GelMA‐A in macrophages, as macrophages are also present in high numbers at the lesion site after SCI and can be targeted to improve recovery.^[^ ^11^, ^12^, ^22^ ^]^ For this purpose, we repeated the above experiments using RAW264.7 macrophages, an immortalized murine macrophage cell line. As expected, LPS stimulation induced a significant increase in TNF‐*α* secretion, and TNF‐*α* and iNOS expression at 24 and 48 h (*p* < 0.001, **Figure** **3**A,B,E–H). LPS stimulation also induced a significant increase in IL‐10 secretion (*p* < 0.0001, Figure 3C,D) and Arg‐1 expression (*p* < 0.01, Figure 3I), highlighting the dynamic nature of immune cell polarization. In contrast to BV2 microglia, there was a significant increase in TNF‐*α* secretion and expression in macrophages that were grown in 3% GelMA‐A at 24 h compared to control cells, potentially due to the measured endotoxin levels described in Section 3.1 (Figure 3A,E). However, TNF‐*α* decreased back to control levels at 48 h (Figure 3B,F). There was no significant difference in the secretion of IL‐10, or the expression of iNOS, CD206 or Arg‐1 between control cells or cells in GelMA, suggesting that 3% GelMA‐A does not affect RAW264.7 macrophage polarization after 48 h.

### 3.4 3% GelMA‐A Does Not Induce a Reactive Response in Astrocytes or Microglia Ex Vivo

**Figure 4:**
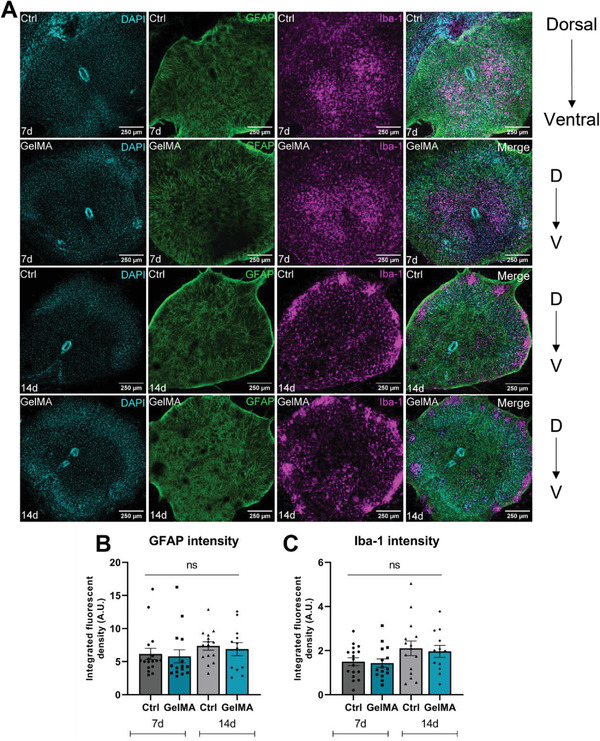
3% GelMA‐A does not significantly affect GFAP or Iba‐1 intensity over 14 days ex vivo. A) Representative photomicrographs showing sum projections of DAPI (cyan), GFAP (green), and Iba‐1 (magenta) in organotypic spinal cord slices from C57BL/6 pups grown on either PET membranes (Ctrl) or on 3% GelMA‐A hydrogels for 7 or 14 days. The orientation of the dorsal‐ventral axis is given on the right of each image panel. Scale bar = 250 µm. Fluorescent density of B) GFAP and C) Iba‐1 staining was quantified using ImageJ. Data are presented as mean ± SEM, *n* = 6 mouse pups per group, 1–4 slices per pup. Analysis by two‐way ANOVA with Tukey's multiple comparisons test, ns *p* > 0.05 compared to control.

While in vitro cell culture models are useful for investigating basic scientific questions, *ex vivo* organotypic cultures are more representative of in vivo tissue architecture, due to preservation of key in vivo tissue characteristics such as multicellular signaling pathways and synaptic activity.^[^ ^80^, ^81^, ^82^ ^]^ Here, we cultured organotypic spinal cord slices isolated from P5‐6 C57BL/6 pups on 3% GelMA‐A and performed immunofluorescent staining of astrocytes and microglia to investigate cellular response ex vivo. In doing so, we have shown for the first time that 3% GelMA‐A hydrogels can support organotypic spinal cord slice culture for up to 14 days (**Figure** **4**A). We found that GFAP staining intensity within the spinal cord slice remained unchanged in slices grown on 3% GelMA‐A versus control slices grown on PET inserts at 7 and 14 days in culture, suggesting 3% GelMA‐A does not increase reactive GFAP expression in astrocytes *ex vivo* (Figure 4B). Similarly, Iba‐1 staining was used to identify microglia within the slice, and we demonstrated that Iba‐1 intensity remained unchanged between 3% GelMA‐A and controls at 7 and 14 days in culture (Figure 4C). Taken together, these results indicate that at 7 or 14 days, 3% GelMA‐A does not cause a change in Iba‐1 or GFAP expression ex vivo, suggesting no direct effect on glial reactivity.

**Figure 5:**
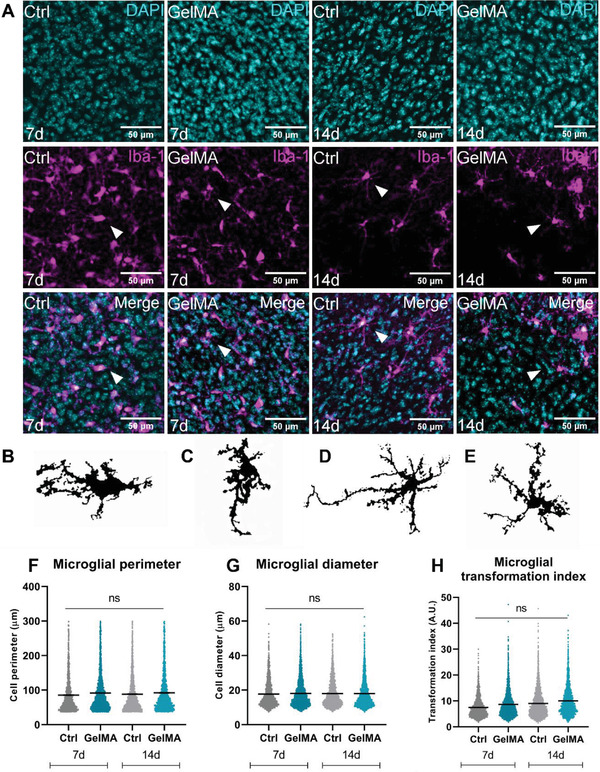
3% GelMA‐A does not significantly affect microglial morphology over 14 days ex vivo. A) Representative photomicrographs showing sum projections of DAPI (cyan) and Iba‐1^+^ microglia (magenta) in organotypic spinal cord slices from C57BL/6 pups grown on either PET membranes (Ctrl) or on 3% GelMA‐A hydrogels for 7 or 14 days. Scale bar = 50 µm. B–E) Ramified microglia highlighted with white arrows are shown in more detail. Quantification of F) microglial perimeter, G) diameter, and H) transformation index was done using ImageJ. Data are presented as mean ± SEM, *n* = 6 pups per group, 1–4 slices per pup. Analysis by two‐way ANOVA with Tukey's multiple comparisons test, ns *p* > 0.05 compared to control.

Higher magnification images of Iba‐1^+^ microglia were then used to investigate microglial morphology as an indicator of microglial activation^[^ ^74^, ^83^ ^]^ (**Figure** **5**). Specifically, microglial perimeter, Feret's maximum diameter and transformation index were measured. A ramified cell with long processes and a relatively small cell body will be represented by a higher transformation index than a rounder, more amoeboid cell with short processes, therefore taking cell size and cell ramification into account. Furthermore, ramified microglia should display larger values for cell perimeter and diameter compared to amoeboid cells.^[^ ^83^, ^84^ ^]^ Figure 5 shows representative immunofluorescence images from control and GelMA organotypic spinal cord slices, with examples of ramified microglia highlighted in Figure 5B–E. There was no significant difference in microglial diameter, perimeter, or transformation index between groups at 7 or 14 days in culture (Figure 5F–H), suggesting that 3% GelMA‐A does not directly affect microglial ramification ex vivo.

## 4 Discussion

A successful biomaterial has several key characteristics—physical properties mirroring the receiving microenvironment, high biocompatibility, and low immunogenicity.^[^ ^31^, ^85^, ^86^ ^]^ While GelMA is widely used across the tissue engineering field,^[^ ^54^, ^57^, ^62^, ^63^, ^65^ ^]^ it's application for preclinical SCI has mainly been limited to therapeutic cell delivery,^[^ ^67^, ^68^, ^69^ ^]^ or as a support matrix to promote axonal regeneration,^[^ ^37^, ^70^, ^71^ ^]^ with little to no evaluation of the effect of GelMA on the secondary neuroinflammatory response reported. Furthermore, several of these applications describe a GelMA‐based scaffold rather than an injectable hydrogel, posing a challenge to clinical translation due to the requirement for surgical implantation into the cord, risking damage to uninjured tissue.^[^ ^37^, ^69^, ^71^ ^]^ Here, we have shown that soft, injectable 3% GelMA‐A hydrogels support microglial survival at a rate of >90% after 7 days, do not directly affect microglial or macrophage polarization in vitro, and support the culture of organotypic spinal cord slices while avoiding glial activation ex vivo. Together, this demonstrates that 3% GelMA‐A hydrogels present a promising biomaterial platform in the specific context of the immune microenvironment after SCI.

We first aimed to identify an optimal GelMA formulation by comparing the stiffness, swelling ratios and cytotoxicity of various (*w/v*) concentrations of hydrogel derived from either gelatin type‐A or type‐B. Neural and glial cells in the CNS exhibit mechanosensitivity with a preference toward “softer” substrates that mimic the mechanical properties of the native microenvironment.^[^ ^46^, ^47^ ^]^ For example, Moshayedi et al. demonstrated that primary rat microglia and astrocytes had reduced inflammatory gene and protein expression when grown on soft substrates (storage modulus ≈100 Pa) compared to stiffer substrates (>10 kPa).^[^ ^47^ ^]^ Additionally, stiffer GelMA hydrogels (storage modulus ≈10 kPa) induced an inflammatory response in mouse bone marrow‐derived macrophages compared to softer GelMA hydrogels (storage modulus <1000 Pa), as determined by increased iNOS expression and decreased Arg‐1 expression in vitro.^[^ ^41^ ^]^ 3% GelMA‐A displayed a storage modulus of 31.4 ± 4.6 Pa (Figure 1B), which is within the above outlined biocompatible range for CNS tissue engineering.^[^ ^41^, ^46^, ^47^ ^]^ Additionally, a porous microstructure should facilitate molecular mobility within the hydrogel network, which is a key property for therapeutic cargo delivery applications (Figure 1A and Figure S2, Supporting Information). 3% GelMA‐A has an average pore size of 105 ± 6.6 µm (Figure S2A, Supporting Information), which matches the desired range of 20–200 µm to facilitate infiltration of neural cells (e.g., oligodendrocytes, 6–9 µm) and neuronal fibers (up to 20 µm), as well as larger therapeutic biomolecules.^[^ ^31^, ^87^, ^88^, ^89^ ^]^ While GelMA's porous structure facilitates molecular and cell mobility through the injected hydrogel,^[^ ^68^, ^70^ ^]^ the injectability of 3% GelMA‐A outlined in Figure 1K introduces the possibility of processing by different techniques, including 3D printing and microfabrication, to achieve an aligned structure to guide regeneration of severed axons in the spinal cord.^[^ ^37^, ^69^, ^71^ ^]^ Furthermore, following SCI, increasing intraspinal pressure can contribute toward secondary injury and thus the swelling capacity of injectable hydrogels should be limited (Figure 1C,D).^[^ ^49^, ^50^ ^]^ We have shown that 3% GelMA‐A is soft, porous and exhibits limited swelling over 14 days, and thus satisfies the biocompatible mechanical properties outlined in Section 1.

We then analyzed BV2 microglial survival within each GelMA formulation over 7 days (Figure 1D–G). Our results show that BV2 survival is higher in GelMA‐A versus GelMA‐B over 7 days. A potential explanation may be the differential charge of GelMA‐A versus GelMA‐B. The hydrolysis of collagen under either acidic or alkaline conditions gives rise to either gelatin type‐A or type‐B, respectively.^[^ ^54^ ^]^ The amino acid residues of gelatin type‐A and type‐B differ due to deamidation of asparagine and glutamine under alkaline conditions, resulting in a higher number of aspartic acid and glutamic acid residues in type‐B versus type‐A. This in turn affects the isoelectric point, which for type‐A remains similar to collagen at pH 8–9, while for type‐B it is at pH 4.8‐5.5.^[^ ^90^ ^]^ The effect of substrate charge on neuronal growth is well established, with positively charged substrates exhibiting improved cell attachment and axonal outgrowth.^[^ ^91^, ^92^, ^93^ ^]^ Hejčl et al. reported that astrocytes preferentially infiltrated negatively charged or neutral hydrogel implants over positively charged hydrogels,^[^ ^93^ ^]^ suggesting that hydrogel charge influences different neural cell types in different ways. While the effect of substrate charge on microglia is not well explored, it has been shown that positive substrates are more cytotoxic to macrophages than negative substrates,^[^ ^94^ ^]^ and thus the differential charge of GelMA‐A versus GelMA‐B may explain the pronounced cytotoxicity of GelMA‐B shown in Figure 1G,H.

Endotoxin content may also contribute to microglial survival rates in GelMA. Endotoxins drive activation of immune cells and thus are an important factor to consider in the context of immunotherapeutic biomaterials for SCI.^[^ ^75^, ^76^ ^]^ While the endotoxin levels reported here exceed the current FDA limit of 0.5 EU mL^−1^,^[^ ^77^ ^]^ the lack of endotoxin‐free raw materials or a designated clean room would make achieving endotoxin levels under this limit difficult in a laboratory environment. We found no difference in the endotoxin content of 3% GelMA‐A versus GelMA‐B hydrogels, while each GelMA hydrogel had a lower endotoxin content than the original corresponding gelatin (Figure 1J). This suggests that endotoxin content is not directly responsible in this case for the differential survival of microglial in GelMA‐B versus GelMA‐A (Figure 1E–H).

After identifying 3% GelMA‐A as the optimal hydrogel formulation in the context of mechanical properties and cytocompatibility, we then determined if 3% GelMA‐A affects immune cell polarization in vitro, using microglia and macrophages since both cell types are present at the SCI lesion post‐injury.^[^ ^9^, ^10^, ^11^ ^]^ Local microglia and infiltrating macrophages exist on a dynamic activation spectrum ranging from a classically activated pro‐inflammatory/“M1‐like” state to an alternatively activated anti‐inflammatory/“M2‐like” state, exerting both detrimental and beneficial effects on recovery, respectively.^[^ ^12^, ^15^, ^16^, ^95^ ^]^ The complexity of immune cell polarization and the dynamic, multidimensional immune states that exist in vivo^[^ ^20^, ^21^, ^96^ ^]^ are illustrated in Figure 3, whereby LPS stimulation induces increased expression of both “M1‐like” markers TNF‐*α* and iNOS, and “M2‐like” marker Arg‐1 in RAW264.7 macrophages. However, the simplified classification offers a useful tool for characterizing the immunotherapeutic complexity in vitro. We did not observe the co‐expression of “M1/M2‐like” markers in BV2 microglia, and BV2 microglia did not secrete IL‐10 at a detectable level (Figure 2). This is consistent with previous literature reporting that IL‐10 secretion from both untreated microglia and BV2 microglia stimulated with 10 µg mL^−1^ LPS was <10 pg mL^−1^.^[^ ^97^, ^98^ ^]^ After 48 h in culture, we found that neither microglia nor macrophages were affected by 3% GelMA‐A (Figures 2 and 3). This is reflected by unaltered expression of proinflammatory M1‐like (TNF‐*α* and iNOS) and anti‐inflammatory M2‐like (Arg‐1 and CD206) markers, and unaltered secretion of TNF‐*α* and IL‐10 compared to control cells. The exception is the significant increase in TNF‐*α* expression and secretion by RAW264.7 macrophages at 24 h (Figure 3A,E). It is possible that this is a rapid, transient “foreign body”‐type response to endotoxins within the hydrogel, or as the macrophages adjust to their 3D growth environment during the initial 24 h, followed by a decrease in TNF‐*α* back to control levels after 48 h (Figure 3B,F). Similar effects have been previously reported, with adhesion‐mediated NF‐*κβ* activation suggested as a potential mechanism for M1‐like macrophage polarization in a 3D environment.^[^ ^42^, ^99^, ^100^ ^]^

Finally, hydrogel biocompatibility was further investigated using an ex vivo organotypic spinal cord slice model. Monolayer 2D culture conditions do not mimic spinal cord tissue biomechanics, leading to loss of polarization and cell diversity. Organotypic slices are a more representative model as they recapitulate in vivo neural tissue architecture by retaining tissue organization and cellular interactions.^[^ ^74^, ^80^, ^81^ ^]^ Glial cells are key orchestrators of the secondary response after SCI, and preservation of glial interactions is critical for achieving a representative ex vivo SCI model.^[^ ^82^, ^101^ ^]^ Organotypic slices allow for analysis of glial cells in a representative environment that avoids the potential confounding effects of isolating the cells for monolayer cultures. Following SCI, the influx of serum components through the compromised BSCB and the production of cytokines by activated microglia and macrophages induce a reactive response in astrocytes at the injury site, in which GFAP expression and proteoglycan deposition is upregulated, forming a fibrous glial scar around the lesion.^[^ ^102^, ^103^ ^]^ Therefore, we used GFAP and Iba‐1; a reactive astrocytic filament protein^[^ ^102^, ^103^ ^]^ and a pan‐microglial marker,^[^ ^104^ ^]^ respectively, to detect astrocytes and microglia in organotypic spinal cord slices prepared from C57BL/6 mouse pups and grown on either PET membranes or on 3% GelMA‐A. We found that 3% GelMA‐A had no effect on GFAP or Iba‐1 expression at 7‐ or 14‐days ex vivo, and in doing so, we have shown for the first time that GelMA hydrogels can be used as substrates to support the culture of organotypic spinal cord slices (Figure 4).

First described by Río‐Hortega in 1919, it is now widely accepted that microglia undergo morphological changes during activation.^[^ ^74^, ^83^, ^84^, ^105^ ^]^ Therefore, we analyzed microglial morphology as an indicator of the cellular activation state. Ramified microglia are generally considered to be in a homeostatic surveillant state, with larger values for cell perimeter, maximum cell diameter and transformation index, while activated microglia can appear as amoeboid or comparatively rounded, with smaller values for these same parameters.^[^ ^74^, ^83^, ^84^ ^]^ We found no difference in the values for these parameters between control and GelMA groups, indicating 3% GelMA‐A does not influence microglial morphology ex vivo. Taken together with the finding that 3% GelMA‐A does not affect Iba‐1 expression, this supports the claim that 3% GelMA‐A does not directly affect microglial activation ex vivo.

## 5 Conclusion

The impact of GelMA hydrogels on the preclinical SCI landscape is more evident now than ever with the number of publications increasing year on year,^[^ ^37^, ^67^, ^68^, ^69^, ^70^, ^71^ ^]^ due to advantageous properties such as injectability, presence of cell attachment motifs, biocompatibility, and biodegradability. Our work contributes to the growing literature on GelMA for SCI repair by establishing minimal immunomodulatory impact of the biomaterial for the first time in a CNS‐specific in vitro and ex vivo environment. We found that microglial survival remained high in 3% (*w/v*) GelMA hydrogels synthesized from gelatin type‐A over 7 days, with no change in expression of polarization markers by microglia or macrophages at 48 h, indicating high cytocompatibility and low immunogenicity in vitro. Additionally, 3% GelMA‐A did not initiate a reactive response in glial cells ex vivo, showing for the first time that 3% GelMA‐A can support organotypic spinal cord slice culture for up to 14 days. Furthermore, our demonstration of LAP as a photoinitiator allows for in situ polymerization with a visible light source, avoiding the need for UV photocrosslinking, which is commonly used for GelMA hydrogels^[^ ^66^, ^68^, ^69^ ^]^ but risks tissue damage due to higher energy radiation.

Additionally, our pared back in vitro*/*ex vivo approach allows for direct translation of our results to a preclinical in vivo environment. The use of in vivo animal models has been critical in revealing underlying mechanisms of SCI pathophysiology.^[^ ^9^, ^106^, ^107^, ^108^ ^]^ Design of therapeutics for preclinical in vivo studies could be guided using the combined in vitro and ex vivo approach developed here, improving the possibilities for translation to clinical SCI. The application of GelMA as an immunotherapeutic platform for SCI is limited, with therapeutic cell delivery and tissue regeneration instead taking the focus.^[^ ^37^, ^43^, ^67^, ^68^, ^70^, ^71^ ^]^ However, cell graft survival and axonal regeneration cannot occur successfully in the harsh SCI immune microenvironment.^[^ ^12^, ^109^, ^110^ ^]^ Therefore, immunomodulation is a critical therapeutic approach that should be incorporated into future biomaterial‐based design strategies.

Given the biocompatible mechanical properties and high cytocompatibility (Figure 1), low immunogenicity (Figures 2 and 3), and the novel ex vivo work demonstrating biocompatibility of 3% GelMA‐A (Figures 4 and 5), we conclude that 3% GelMA‐A is an immunologically inert hydrogel upon which an immunotherapeutic system can be built, e.g., through the incorporation of immunomodulatory biomolecules or cells, thus facilitating a permissive proregenerative environment that can ultimately improve recovery after SCI.

## Conflict of Interest

The authors declare no conflict of interest.

## Supporting information

Supporting Information

## Data Availability

The data that support the findings of this study are available from the corresponding author upon reasonable request.
