# Supplementary material for: An In Vitro and Ex Vivo Analysis of the Potential of GelMA Hydrogels as a Therapeutic Platform for Preclinical Spinal Cord Injury
Source: Adv Healthc Mater. 2023 May 12;12(26):2300951. doi: 10.1002/adhm.202300951 (PMC11468190; doi:10.1002/adhm.202300951)
Supplement: Supplementary file 1 — Supporting Information [file ADHM-12-2300951-s001.pdf]

ADVANCED HEALTHCARE MATERIALS

Supporting Information

for *Adv. Healthcare Mater.*, DOI 10.1002/adhm.202300951

An In Vitro and Ex Vivo Analysis of the Potential of GelMA Hydrogels as a Therapeutic Platform for Preclinical Spinal Cord Injury

*Ciara M. Walsh, Jacek K. Wychowaniec, Louise Costello, Dermot F. Brougham and Dearbhaile Dooley**

Supporting Information

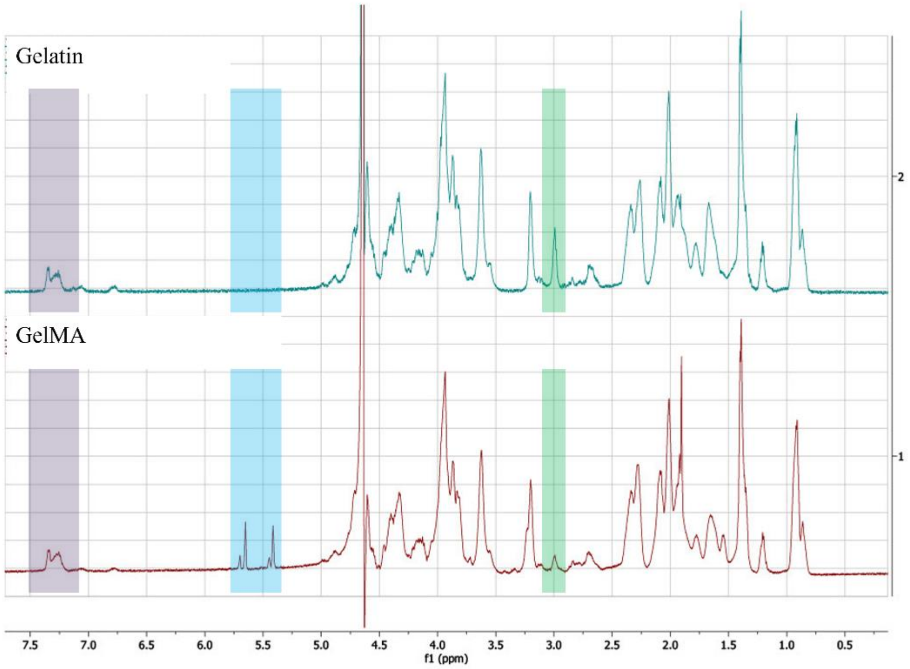

Figure S1: Representative ^{1}H NMR spectra of unmodified gelatin and GelMA. *Highlighted regions were used for calculating DoM, with; purple aromatic amino acids resonances (7.1-7.5 ppm) that are not chemically modified; blue methacrylate vinyl groups that appear after reaction (5.3-5.6 ppm), and; green lysine amino acids (2.9-3.0 ppm).*

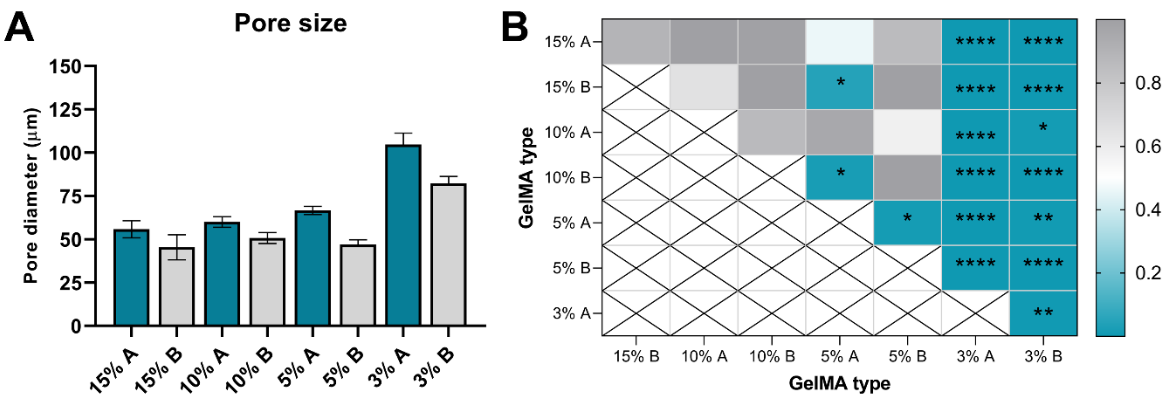

*Figure S2: Quantification of GelMA hydrogel pore size. (A) Pore size of GelMA hydrogels calculated from SEM images. (B) Results of 2-way ANOVA with Tukey's multiple comparison test comparing pore sizes in different hydrogels. Results are displayed as mean ± SEM of 41-206 measured pores from each hydrogel sample. *p < 0.05, **p < 0.01, ****p < 0.0001.*

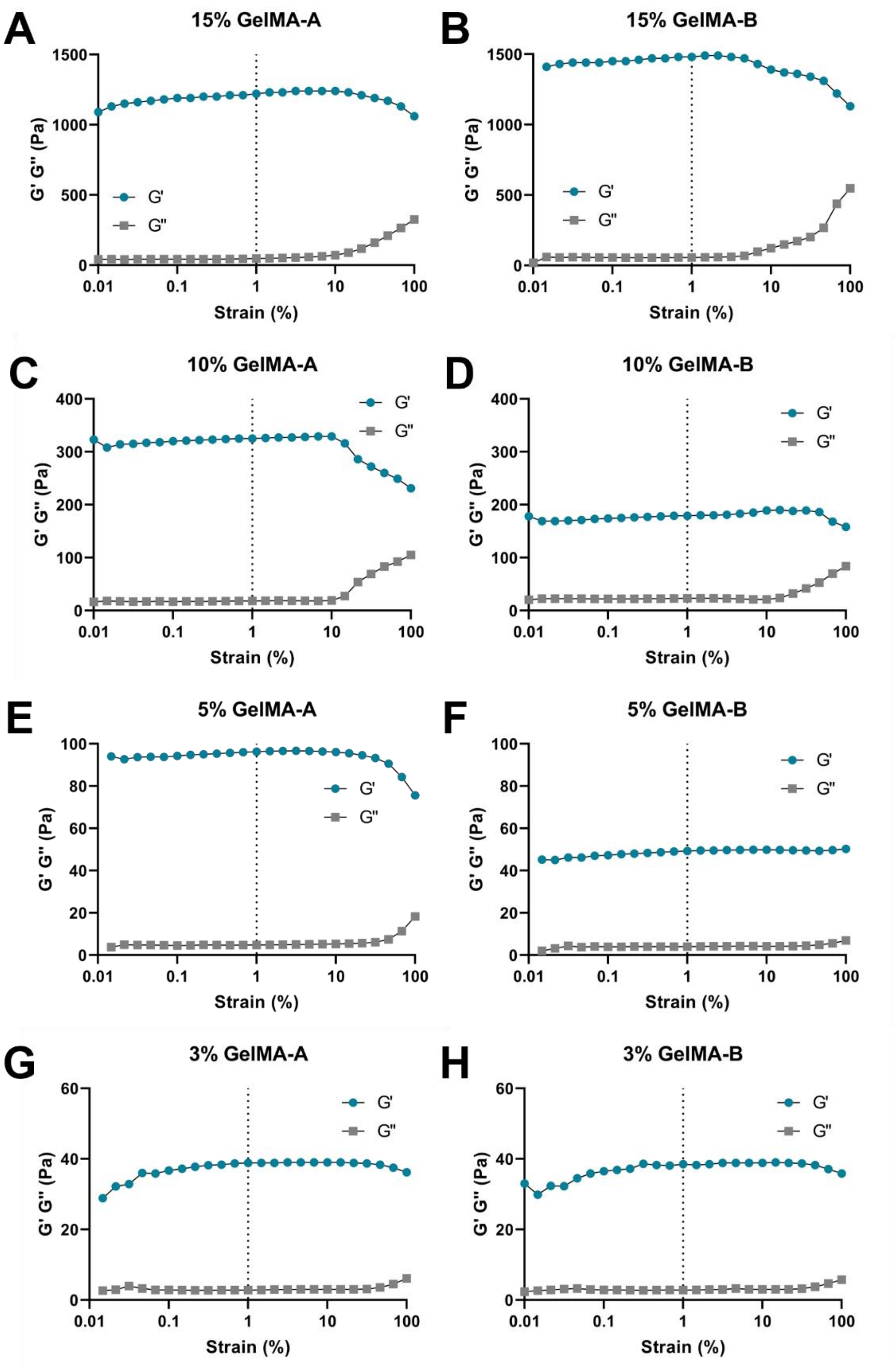

Figure S3: Representative amplitude sweep showing storage (G') and loss (G'') moduli measured at 1 Hz and strain increasing from 0.01% to 100% at 37°C to determine the linear viscoelastic region (LVR) to be applied for subsequent frequency sweep measurements.
